# Fifty Years of Research on European Mink *Mustela lutreola* L., 1761 Genetics: Where Are We Now in Studies on One of the Most Endangered Mammals?

**DOI:** 10.3390/genes11111332

**Published:** 2020-11-11

**Authors:** Jakub Skorupski

**Affiliations:** 1 Institute of Marine and Environmental Sciences, University of Szczecin, Adama Mickiewicza 16 St., 70-383 Szczecin, Poland; jakub.skorupski@usz.edu.pl; Tel.: +48-914-441-685; 2 Polish Society for Conservation Genetics LUTREOLA, Maciejkowa 21 St., 71-784 Szczecin, Poland

**Keywords:** conservation genetics, cytogenetics, endangered species, genetic markers, genomics, mitogenomics, *Mustela* sp., Mustelidae, phylogenetics, population genetics

## Abstract

The purpose of this review is to present the current state of knowledge about the genetics of European mink *Mustela lutreola* L., 1761, which is one of the most endangered mammalian species in the world. This article provides a comprehensive description of the studies undertaken over the last 50 years in terms of cytogenetics, molecular genetics, genomics (including mitogenomics), population genetics of wild populations and captive stocks, phylogenetics, phylogeography, and applied genetics (including identification by genetic methods, molecular ecology, and conservation genetics). An extensive and up-to-date review and critical analysis of the available specialist literature on the topic is provided, with special reference to conservation genetics. Unresolved issues are also described, such as the standard karyotype, systematic position, and whole-genome sequencing, and hotly debated issues are addressed, like the origin of the Southwestern population of the European mink and management approaches of the most distinct populations of the species. Finally, the most urgent directions of future research, based on the research questions arising from completed studies and the implementation of conservation measures to save and restore *M. lutreola* populations, are outlined. The importance of the popularization of research topics related to European mink genetics among scientists is highlighted.

## 1. Introduction

Due to ongoing population depletion, both in terms of the actual number of individuals and area occupied, European mink *Mustela lutreola* L., 1761 is considered one of the most endangered mammalian species in the world [1,2]. The species was originally spread over most of continental Europe, but nowadays only three wild, isolated, declining populations occupying less than 3% of the former range survive [3]. About 5000 individuals are estimated to persist in the wild [3]. Reintroduced populations were established in Estonia and Germany [2]. The main cause of the situation of this species is habitat loss and fragmentation, overhunting, and the effects of introduced invasive American mink *Neovison vison* [4]. The alarming situation of the species is proven by its categorization as critically endangered (CR) by the International Union for Conservation of Nature (IUCN) Red List of Threatened Species, and it is listed in Annex II to the Bern Convention on the Conservation of European Wildlife and Natural Habitats, Annexes II and IV (priority species) of the Council Directive 92/43/EEC on the conservation of natural habitats and of wild fauna and flora, and in The Carpathian List of Endangered Species (critically endangered species (CR)) [2,5].

Despite this, the number of studies on European mink, reflected in the number of scientific papers devoted to the species, is relatively low. The digital repository of the National Centre for Biotechnology Information (NCBI) records less than 60 scientific articles devoted to various aspects of the biology of the species. In comparison, the same repository lists as many as 2000 articles related to the giant panda *Ailuropoda melanoleuca* [6]. Studies in the field of genetics of *M. lutreola* are limited and urgently need to be completed, especially in the context of the progressing extinction process and the disappearance of its numerous populations in France, Belarus, and Russia, among others [2]. Among the scientific articles devoted to the species and recorded in the NCBI repository, only ~15 concern genetic issues (not including multispecies phylogenetic analyses not directly focused on European mink) [6]. The rapidly shrinking and vanishing genetic resources will largely never be studied and described, which is an irreversible loss from cognitive and practical points of view [7,8]. The meagre data on interpopulation genetic diversity may significantly impair the efficacy of the implemented activities for restitution of the European mink, especially in the context of conservation breeding and species reintroduction [7,9,10,11,12].

Notably, only (conservation) genetics can provide tools to rescue species affected by the extinction vortex [13], which, in turn, requires more research initiatives in the conservation genetics of the European mink [14,15].

The pioneer genetic studies on European mink concerning cytogenetics were conducted in the former USSR. To date, works by Volobuev and Ternovsky [16], Volobuev et al. [17], Graphodatsky et al. [18], Graphodatsky et al. [19], and Graphodatsky and Radjabli [20], strongly affiliated with the Siberian Branch of the Russian Academy of Sciences in Novosibirsk (Russia), are the primary sources of information on *M. lutreola* karyotype. Further research on the genetics of European mink relates primarily to genetic markers [9,21], the phylogenetic relationships of the species [22,23,24,25,26], noninvasive methods of identification [27], assessment of intraspecies genetic diversity [8,14,28,29,30], and mitochondrial DNA (mtDNA) studies [24,28,31,32]. Worth mentioning are studies concerning molecular ecology on issues relating to European mink and implementing genetic research methods [33,34,35,36].

The purpose of this review was to present the current state of knowledge about the genetics of European mink, with particular emphasis on the possibility of its practical application in species conservation. The case of *M. lutreola* provides a good example of the possibility of achieving the goal of conservation genetics, i.e., applying genetic knowledge and methods to preserve endangered species, as well as to support evidence-informed conservation activities and strategies based on knowledge about the current state of the species’ genetic resources [32,37].

The added value of this review article is an extensive and up-to-date review of the available specialist literature on the genetics of European mink, including an update detailing in terms of genetics, the species bibliography published by Youngman [38]. The review not only includes articles published in scientific journals and books, but also in scientific conference reports, Ph.D. theses, and websites of research and nature conservation institutions. For the phylogeographic and demographic studies, only those focused on population genetics of contemporary populations and reasons for their current geographical distribution are included in this review.

Additionally, a definition of conservation (captive) breeding genetics is formulated.

## 2. Cytogenetics

The diploid number of the chromosomes of European mink is 38, which is typical for many species of the Mustelidae Fischer, 1817 family; for more than 60% of species of this group, 2*n* = 38 [39,40]. Among the representatives of the *Mustela* L., 1758 genus, the diploid number of chromosomes ranges from 38 to 44 [41,42]. Significantly, in the case of American mink, 2*n* = 30, which, on a cytogenetic basis, additionally indicates a relatively low degree of its evolutionary relationship with European mink [43].

The chromosomal set of *M. lutreola* consists of five pairs of metacentric chromosomes of different sizes, two pairs of subtelocentric chromosomes, five pairs of submetacentric chromosomes, and seven pairs of telocentric chromosomes [16]. The X chromosome is submetacentric, while the Y chromosome is metacentric [18]. As with other representatives of the Mustelidae family, the Y chromosome is the smallest one [40]. The fundamental number (*FN*, number of chromosomal arms) is 62, while the fundamental autosomal number (*FNa*, number of autosomal arms) is 58 [16]. Nucleolus organizer regions (NORs) were identified in one pair of telocentric chromosomes [16]. The standard pattern of European mink karyotype is not currently established [40].

The characteristics of the G and C bands of chromosomes of European mink were presented by Graphodatsky et al. [18,19,44]. The pattern of the Ag-NOR bands, obtained by silvering the nucleolar organizer regions, was also described [20]. A detailed comparison of the patterns of G bands of chromosomes of European mink, American mink, least weasel *Mustela nivalis*, mountain weasel *Mustela altaica*, Japanese marten *Martes melampus*, European badger *Meles meles*, and striped polecat *Ictonyx striatus* was presented by Graphodatsky et al. [44]. In terms of the G-band pattern and chromosomal number, size, and morphology, European mink shows many similarities with the Siberian weasel *Mustela sibirica* [18,23].

## 3. Genetic Markers

The best-known genetic markers of European mink are the microsatellite nuclear sequences (short tandem repeats (STRs) and simple sequence repeats (SSRs)), which are sequential patterns of DNA consisting of several nucleotides and tandem repeats [21]. They are used in phylogenetic studies and analyses of intra- and interpopulation genetic variation and internal genetic structure for detection of evolutionary events in phylogenesis of European mink, phylogeographic reconstructions, and potentially identification of interspecies *M. lutreola* × *Mustela putorius* hybrids [9,30,45,46,47]. The names of microsatellite markers of European mink consist of a unique number preceded by the abbreviation “*Mlut*”. Cabria et al. [9] identified eight unique microsatellite loci of *M. lutreola*, which are listed in Table 1.

Microsatellite markers of European mink were successfully amplified in other species of the Mustelidae family, including in *Mustela eversmanii*, *Mustela putorius furo*, *M. sibirica*, *M. nivalis*, *N. vison*, *Mustela erminea*, *Martes martes*, and *Martes foina*, among others [9,48], demonstrating the possibility of using of the STR markers of *M. lutreola* in studies on genomes of other mustelids. In turn, Peltier and Lodé [45], Michaux et al. [8], Lodé et al. [49], and Cabria et al. [30] positively assessed the possibility of using starter sequences developed for amplification of microsatellites in the genome of other species of the Mustelidae family for studies on the population genetics, phylogenetics, and phylogeography of European mink (Table 2).

Besides the polymorphism of neutral genetic markers, variants of allozymes and the *drb* gene from the family of genes of major histocompatibility complex (MHC) class II were analyzed in *M. lutreola* [49,50,51,52]. Of 36 allozyme loci of European mink analyzed by Lodé et al. [49], two allelic forms were determined for only four of them (the gene for carboxylesterase/EC 3.1.1.1, *est-2*; the gene for NADP-dependent cytosolic malate dehydrogenase/EC 1.1.1.40, *me−1*; the gene for malate dehydrogenase (MDH)/EC 1.1.1.37, *mdh−1;* and the gene for nonspecific protein). All other loci were monomorphic, whereas for samples analyzed in parallel from the European polecat, nine polymorphic loci were found [49]. For the *drb* gene, Becker et al. [50] described nine allelic forms. Nishita et al. [51,52] conducted phylogenetic analyses based on the sequence of the second exon of the *drb* gene, demonstrating the evolutionary closeness of this sequence in *M. sibirica*, *Mustela itasi,* and *M. lutreola* and its trans-species polymorphism (TSP), indirectly evidencing selection balance.

## 4. Genomics

The genome of European mink has not yet been sequenced. However, Mouton et al. [53] reported the first attempt of whole genome sequencing (30×) of one individual from the Charente-Maritime region (Southwestern France) to perform a genome scan for runs of homozygosity (ROH) to estimate inbreeding, as well as to investigate recent demographic events on the basis of genomic data.

For the species, 160 nucleotide sequences (DNA and RNA) were deposited in GenBank [54]. This number includes the complete sequence of the nuclear gene for the angiotensin-I-converting enzyme 2 (ACE2) of 4097 bp, 61 records for mtDNA, 30 of which represent haplotypes of the *cytb* gene (26 for fragments of 337 to 504 bp and four for the complete gene sequence of 1140 bp), 23 haplotypes for the control region (357 to 990 bp), and the whole mitogenome sequence. In comparison, 357,611 and 19,698 records of the nucleotide sequences of *M. putorius* and *M. putorius furo* [54] and *N. vison* were deposited in GenBank, respectively.

Lushnikova et al.’s [55] work can be considered the first genomic research on European mink. Their studies concerned DNA reassociation kinetics, revealing the share of the genomic DNA fraction representing repetitive sequences in the species genome. The genome size of *M. lutreola* was estimated at 6.4 pg, and the shares of rapidly (representing highly repetitive DNA sequences), intermediately (representing moderately repetitive sequences), and slowly- (representing low-repetitive, complex, and unique DNA sequences) renaturing fractions were shown to be 17%, 13%, and 70%, respectively [55]. In the same study, differences in DNA quantity and heterochromatin amount were found between European mink, American mink, and marbled polecat *Vormela peregusna* [55]. The most variable component in the mentioned species is the moderately repetitive genome component [55].

Pioneering studies on the genome scale included comparative studies concerning RFLP-*Eco*RI and RFLP-*Bam*HI polymorphisms in *M. lutreola*, *M. putorius*, *N. vision*, *M. erminea*, *M. sibirica*, and *V. peregusna* [56]. The obtained digest patterns demonstrated interspecific variation in length and the number of a repeated sequences copies. In European mink, the identified 0.7 kb *Eco*RI repeats were dispersed over karyotype, the 1.9 kb *Bam*HI repeats were concentrated in the heterochromatic pericentromeric regions and additional chromosome arms, while the 1.35 kb *Bam*HI repeats were only located in the centromeric regions [56]. The *Bam*HI repeats (interspecific variability of DNA–DNA hybridization patterns) were also applied to consider the phylogenetic relationships of the abovementioned species, indicating existence of a common evolutionary group including *M. lutreola*, *M. sibirica*, and *M. putorius* [57].

Due to its high cytogenetic similarity and proven close phylogenetic relationships [22,24,34,42], the size of the nuclear genome of European mink can be estimated on the basis of the sequenced genome of the ferret (MusPutFur1.0, RefSeq assembly accession: GCF_000215625.1) and *M. putorius* (polecat_10x_lmp_bionano, GenBank assembly accession: GCA_902207235.1) as being about 2.411–2.474 million bp, the content of GC pairs as about 42%, and the number of genes as about 27,300 [58].

The complete reference mitochondrial genome of European mink was sequenced de novo in 2017 (GenBank accession code: MT304869), with the length of nucleotide sequence being 16,523 bp [32]. The comparison of the recognized sequence of mitogenome of *M. lutreola* with the complete sequences of mitochondrial genomes of 24 Mustelidae species deposited in GenBank and conducted in BLAST [59] showed a similarity at the level of 86–99% (Table 3). The phylogenetic analysis conducted on the basis of the recognized sequence of *M. lutreola* mtDNA indicated its high affinity with European polecat (and ferret) and its explicit presence in the clade including *M. eversmanni*, *Mustela nigripes*, *M. sibirica*, and *M. itatsi* [60]. Comparison of the mitogenome sequence of European mink and European polecat (GenBank accession code: KT693383) showed a discrepancy of 158 single-nucleotide differences [60].

## 5. Identification by Genetic Methods

Identification of European mink from environmental and noninvasively obtained samples (e.g., hair follicles, feces, and environmental DNA) is important for species in situ conservation efforts [27,46,61,62,63]. Its distinction from sympatric (or parapatric) *N. vison* and *M. putorius*, occurring in the same areas, enables the effective control of the population of the former abovementioned invasive alien species that are dangerous for European mink, and allows the assessment of the possible occurrence and scale of hybridization with the latter species [27,46,47,64,65,66,67].

An example is the genetic test for the distinction of European mink, European polecat, and American mink, developed by Gómez-Moliner et al. [27]. The proposed protocol is based on the nested-PCR of the fragment of the mitochondrial control region (D-loop) sequence, followed by digestion of the resulting amplicons (240 bp in case of all three species) with a mixture of restriction enzymes *Rsa*I and *Msp*I [27]. The obtained restriction patterns enable the differentiation of both species, discriminating two haplotypes characteristic of *M. lutreola*, two other haplotypes characteristic of *M. putorius*, and one haplotype characteristic of *N. vison* [27]. The advantage of this method is that it was designed for the use of small amounts of degraded DNA obtained from fecal samples [27].

Another noninvasive method for the identification of European mink, also differentiating it from European polecat and American mink, was developed by López-Giráldez et al. [46]. This method is based on an amplification of the species-specific nuclear microsatellite sequence *Mel*08, according to the procedure described by Domingo-Roura [68]. At the stage of evaluation of the length of the amplification products, *M. lutreola* and *M. putorius* (221 bp product for both species) can be distinguished from *N. vison* (436 bp), whereas the use of digestion with restriction enzyme *Aci*I can distinguish European mink (no digestion occurs) from European polecat (digestion products of 7, 78, and 136 bp) [46]. The advantage of this method is its simplicity and low cost, and its applicatory value is highlighted by the possibility of identifying species that often coexist and belong to the same ecological guild in Europe (semiaquatic carnivorans), despite a totally different approach (control and eradication of the invasive alien population of *N. vison* vs. urgent conservation efforts toward *M. lutreola*) [27,46]. Collecting the genetic material for the abovementioned tests can be noninvasive by using hair traps for sampling hair with hair follicles [46].

Oliveira et al. [61] developed a molecular test with high discriminatory power based on the polymorphisms detected in a nuclear interphotoreceptor retinoid-binding protein (*irbp*; 221 bp fragment of exon 1), arguing that identification methods based on mtDNA are subject to risks from nuclear insert copies, high intraspecific diversity, and heteroplasmy. The distinction between European mink, European polecat, and American mink is based on the differentiation of species-specific single-strand conformation polymorphism (SSCP) electrophoretic patterns [61]. The PCR-SSCP method was optimized for scat and hair samples [61]. Comparing species-distinguishing methods using nuclear DNA and mtDNA, the use of only the latter is highly limiting in cases of natural hybridization, which occurs between *M. lutreola* and *M. putorius* [49,69].

Kiseleva and Sorokin [70] performed detection of European mink in Chelyabinsk oblast and the Republic of Bashkortostan (Russia) using noninvasive DNA sampling from feces. Individuals of *M. lutreola* were detected by the DNA barcoding method following the protocol developed by Fernandes et al. [71], which is based on two sets of species-specific primers targeting the cytochrome *b* gene (*cytb*) sequence (*Mlutreola* F1/5′-AGCTCATCAACAACTCAC-3′ and *Mlutreola* R1/5′-CCATAGTTGACGTCTCGA-3′, amplicon length of 193 bp; and *Mlutreola* F1/5′-AGCTCATCAACAACTCAC-3′ and *Mlutreola* R1b/5′-CCATAATATAAACCCCGC-3′, amplicon length of 280 bp).

## 6. Population Genetics and Phylogeography

Studies on the population genetics of *M. lutreola* focused on defining genetic diversity between preserved populations of the species. Analyses of the intraspecies genetic structure of European mink showed its relatively high genetic diversity, especially in comparison with other Mustelidae taxa [72,73,74,75,76,77,78,79]. However, this diversity is not homogeneous, and various populations show significantly different levels of genetic diversity [30].

The studies of Michaux et al. [7] on the interpopulation analysis of genetic diversity of European mink were based on the complete sequence of the mitochondrial D-loop and 450 bp fragment (5′-region) of the *cytb* gene in 43 individuals: 14 from Russia and Belarus, 2 from Romania, and 27 from France and Spain, with 11, 2, and 1 haplotypes identified for these populations, respectively [7]. The nucleotide diversity (*π*) and haplotype diversity (*h*) [80] were highest in Northeastern Europe (0.0197 ± 0.0025 and 0.978 ± 0.035, respectively), lower in the Romanian population (0.0039 ± 0.0019 and 1, respectively), and the lowest in Southwestern Europe (0 for both indicators) [7].

In a study conducted in 2005, 15 haplotypes for the complete D-loop were identified in the Russian–Belarussian population (18 individuals examined), four in the Romanian population (34 individuals examined), and only one in the French–Spanish population (124 individuals examined) [8]. The nucleotide diversity and haplotype diversity were 0.012 ± 0.0014 and 0.939 ± 0.058 for the Russian–Belarussian population, 0.0012 ± 0.0003 and 0.469 ± 0.088 for the Romanian population, and 0 and 0 for the French–Spanish population, respectively [8]. In the same study, 155 European mink (112 representing the population from Western Europe, 25 from Southeastern Europe, and 18 from Northeastern Europe) were genotyped using five microsatellites (Table 4). Gene diversity (*H_E_*) and allelic richness (*R_S_*) were calculated as 0.539 and 3.76 for Northeastern Europe, 0.458 and 2.89 for Southeastern Europe, and 0.379 and 2.12 for Western Europe, respectively [8]. Microsatellite data revealed that isolation by distance occurs in the Western population, resulting in inbreeding [8]. Multilocus *F_IS_* (inbreeding coefficient) values reached 0.084 in the French–Spanish population, 0.085 in the Romanian population, and 0.182 in the Russian–Belarusian population [8]. The calculated value of the mean kinship coefficient between nearby individuals (*F_ij_*) in the population from West Europe was 0.08 (for distances of <10 km), suggesting mating occurred locally [8]. The obtained results agreed with the outcome of mtDNA analysis, suggesting that the Southeastern and possibly the Western populations underwent a recent bottleneck [8].

An important outcome of the research of Michaux and colleagues [7,8], as well as further studies of Cabria et al. [30] is the identification of three genetically distinguishable extant populations, i.e., the Northeastern European (inhabiting the Volga and the Dvina basin in Russia), the Western European (inhabiting the Southwestern part of France, as well as Northern and Western parts of Spain), and the Southeastern European (inhabiting the Danube Delta in Romania) populations. This conclusion is supported by the results of intraspecific genetic structure analysis, indicating statistically significant pairwise values of fixation indexes [81,82]: *F_ST_* (0.10 between SE and NE Europe, 0.29 between W and SE Europe, and 0.26 between W and NE Europe), *G_ST_* (0.89 between SE and W Europe, 0.54 between W and NE Europe, and 0.42 between NE and SE Europe), and *Φ_ST_* (0.91 between SE and W Europe, 0.71 between W and NE Europe, and 0.26 between NE and SE Europe) [8]. Michaux et al. [8] claimed that the abovementioned values of fixation indexes prove a weak phylogeographical structure for European mink.

Relatively high genetic diversity in the Eastern European population was confirmed by Korablev et al. [83], who examined 11 individuals from the Tver region (Russia). Genotyping based on the 526 bp fragment of the mtDNA D-loop revealed the presence of eight haplotypes, with the calculated values of the indices of nucleotide and haplotype diversity as follows: *π* = 0.0092 ± 0.0055 and *h* = 0.95 ± 0.054 [83]. Analysis involving additional sequences, described by Michaux et al. [7] (Table 4), showed notably increased values of these indices, i.e., *π* = 0.0134 ± 0.0074 and *h* = 0.98 ± 0.027 [83]. European mink is characterized by a higher level of intrapopulation genetic variation than the European polecat from the same region (*π* = 0.0026 ± 0.0019 and *h* = 0.74 ± 0.052) [84]. The difference in the values of genetic diversity parameters in both species can be explained by species-specific ecological and biological features, as well as by differences in population history in a given area [83]. An important conclusion from these studies is that local extinction in the Tver region is not a consequence of genetic decline manifested by a reduction in genetic diversity below the critical level or inbred depression [83].

**Table 4:** Genetic markers used in research on *M. lutreola* population genetics.

Study	Genetic Markers
Michaux et al. [8]	*Mvi*072, *Mvi*075, *Mer*009, *Mer*022, *Mer*41
Korablev et al. [83]	GenBank accession codes: AJ548805-AJ548807, AJ548812, AJ548814, AJ548817
Cabria et al. [30]	*Mlut*04, Mlut20, *Mlut*25, *Mlut*32, *Mlut*35, *Mer*09, *Mer*22, *Mer*41, Mvi022, Mvi072, Mvi075
Lodé [14]	fragments of the genes for AAT-1 and AAT-2—E.C. 2.6.1.1, ACO-1 and ACO-2—E.C. 4.2.1.3, ADA E.C. 3.5.4.4, AK E.C. 2.7.4.3, CK-1 and CK-2—E.C. 2.7.3.2, DDH-1 and DDH-2—E.C. 1.8.1.4, EST-1 and EST-2—E.C. 3.1.1.1, FUMH—E.C. 4.2.1.2, Gly2DH—E.C. 1.1.1.29, G6PDH—E.C. 1.1.1.49, GPI—E.C. 5.3.1.9, HK-1, HK-2 and HK-3—E.C. 2.7.1.1, IDH-1 and IDH-2—E.C. 1.1.1.42, LDH-1 and LDH-2—E.C. 1.1.1.27, MDH-1 and MDH-2—E.C. 1.1.1.37, ME-1 and ME-2—E.C. 1.1.1.40, MPI—E.C. 5.3.1.8, PEP-1 and PEP-2—E.C. 3.4.11.1, PGDH—E.C. 1.1.1.44, PGM-2—E.C. 2.7.5.1, PNP—E.C. 2.4.2.1, SDH—E.C. 1.1.1.14, SOD—E.C. 1.15.1.1, TPI—E.C. 5.3.1.1, and two non-specific proteins
Peltier and Lodé [45]	*Mvi*002, *Mvi*020, *Mvi*072, *Mvi*389, *Mvi*1843, *Mvi*054, *Mvi*111, *Put*FK1
Lodé et al. [49]—population genetics	*Mvi*002, *Mvi*020, *Mvi*027, *Mvi*054, *Mvi*072, *Mvi*075, *Mvi*099, *Mvi*111, *Mvi*389, *Mvi*1843, *Put*FK1
Lodé et al. [49]—*M. lutreola* × *M. putorius* hybrids	allozymic *loci*: *Ada*, *Est-2*, *Mdh-1*, *Me-1*, *Pep-2*, microsatellite *loci*: *Mvi*002, *Mvi*020, *Mvi*075, *Mvi*1843
Cabria et al. [47]	*Mlut*04, *Mlut*20, *Mlut*25, *Mlut*27, *Ml*ut32, *Mlut*35, *Mvi*22, *Mvi*72, *Mvi*75, *Mvi*99, *Mer*09, *Mer*22, *Mer*41

Cabria et al. [30] focused on 11 microsatellite loci (Table 4) and the 614 bp mtDNA fragment including the 3′-end of the *cytb* gene and the control region of 344 individuals. The established parameter values for evaluating the genetic diversity for microsatellite loci are summarized in Table 5; the sequence variability of mtDNA (analyzed in 157 specimens) ranged from high for the Northeastern population (*π* = 0.004 ± 0.003, *h* = 0.862 ± 0.016, 13 haplotypes, 92.3% of private haplotypes) and moderate for the Southeastern population (*π* = 0.0019 ± 0.0015, *h* = 0.352 ± 0.0103, four haplotypes, 75% of private haplotypes), to the lowest for the Western population (only a single haplotype detected). The nucleotide and haplotype diversities calculated for the whole examined group were 0.005 ± 0.003 and 0.857 ± 0.014, respectively [30]. Analysis of the genetic structure of the remaining populations of *M. lutreola* revealed significant geographic structuring (pairwise *Φ_ST_* values calculated based on mtDNA variability ranged from 0.586 to 0.879, whereas the mean *F_ST_* for microsatellite data was 0.224). The presented results indicated higher overall genetic polymorphism and structure in the Northeastern population, slightly lower values in the Romanian population, and significantly lower values in the Western population [30].

Studies conducted by Davison et al. [28] and Cabria [29] led to similar conclusions regarding the genetic diversity of the three abovementioned populations of European mink. Davison et al. [28] identified four haplotypes of the 337 bp fragment of the mitochondrial cytochrome *b* gene in the Eastern European population (30 individuals examined) and two in the Spanish population (7 individuals examined). The same study revealed the existence of four different haplotypes in both the Eastern European and Spanish populations of *M. putorius*.

The cause of high genetic homogeneity of populations living in France and Spain is attributed to the bottleneck and founder effects that might have occurred (one or several times) in the relatively recent past, as well as to the limited gene flow, thereby altering reproductive exchanges [8,14,30]. According to these findings, the population of France and Spain originated in Northern France at the beginning of the 19th century from a few specimens separated from other European populations [8]. The founders probably came from an ancestral Eastern population (encompassing both the Northeastern and the Southeastern genetic pools) during a period of population admixture [30], whereas recolonization after the last glaciation probably occurred from a single refugium supposedly located in Eastern Europe or Asia [7,8,28]. The results of large-scale genetic research by Cabria et al. [30] supported the scenario of stable populations of European mink during Late Pleistocene climate oscillations and expansion along rivers following the last glaciation period, further local extinctions in central Europe, and recent bottleneck events throughout Europe.

These conclusions were also supported by results of genetic polymorphism analysis of 38 genes’ loci (Table 4) conducted on 12 animals from Western France [14]. Of the used loci, only four (*Est−2*, *Mdh−1*, *Me−1,* and *Pnp*) were found to be polymorphic (*H_O_* = 0.09, 0.08, 0.10, and 0.50, respectively; *H_E_* = 0.37, 0.08, 0.48, and 0.52, respectively), with observed heterozygosity averaging 0.02, an expected heterozygosity of 0.038, and an *F_IS_* of 0.48 [14]. In European mink, only 10.5% of loci were shown to be polymorphic; in the European polecat population from Western France (*N* = 49), the polymorphism reached 25.8% and the observed heterozygosity levels averaged 0.057 [85].

Eight microsatellite loci (Table 4) in 12 individuals of *M. lutreola* from the Poitou-Charentes region (Western France), conducted by Peltier and Lodé [45] were genotyped to survey the genetic diversity and population history and confirm the heterozygote deficit resulting from inbreeding (*F_IS_* = 0.1907). The authors suggested that a significant heterozygote deficit in the Southwestern population of European mink was associated with depletion of genetic diversity, which is characteristic of species near extinction.

The level of genetic polymorphism in the French population (Southwestern France) was also studied by Lodé et al. [49]. Genetic variation was measured from 38 gene loci described by Lodé [14] and 11 microsatellite markers (Table 4) in 51 individuals. Only four (10.5%) allozyme loci (*Est−2*, *Mdh−1*, *Me−1*, and *Pnp*) and four (36.4%) microsatellite loci (*Mvi*027, *Mvi*072, *Mvi*1843, and *Put*FK1) were found to be polymorphic. The effective number of microsatellite alleles per locus was estimated at 1.45 and mean observed heterozygosity was 0.095 [49]. In this case, the level of heterozygosity in European mink was significantly lower than that observed in European polecat (*H_O_* = 0.246, *N* = 114), according to parallel analysis [45].

High phenotypic diversity, expressed in the postulated distinction of six [86] to seven [87] subspecies of European mink (*Mustela lutreola lutreola* L., 1761, *M. l. biedermanni* Matschie, 1912, *M. l. binominata* Ellerman and Morrison-Scott, 1951, *M. l. cylipena* Matschie, 1912, *M. l. novikovi* Ellerman and Morrison-Scott, 1951, *M. l. transsylvanica* Éhik, 1932, and *M. l. turovi* Kuznetsov and Novikov, 1939), was not confirmed by intraspecies genetic diversity studies. In the view postulated by Cabria et al. [30], populations of European mink throughout most of the species evolutionary history formed a panmictic continuum. The reason for the observed genetic differentiation between the Russian and Spanish–French populations was suggested to be a recent, human-induced, distance isolation [30]. Currently, recorded morphological differences between populations (e.g., more frequent occurrence of a white patch on the chest for individuals from Eastern Europe) are thought to be an effect of genetic drift [12,30].

Notably, knowledge about the origin of certain populations derived from genetic data affects their management and conservation. An example illustrating this is the lively debate on the controversial natural (colonization) or human-induced (introduction) origin (as suggested by Michaux et al. [7,8] and Cabria et al. [30]) of the French–Spanish European mink population [3,11,88,89,90,91,92,93].

## 7. Phylogenetics

The results of pioneering molecular research on European mink phylogeny based on mitochondrial sequence (*cytb*, *12S* rRNA and the 5′-fragment of the D-loop) were reported by Davison et al. [94]. These results suggested a close evolutionary relationship between *M. lutreola* and polecats (*M. putorius* and *M. eversmanii*, as well as *M. nigripes,* though less expressed), and its more distant relationships with *M. sibirica* and *M. itatsi* [94]. Further analysis based on the *cytb* gene and the D-loop sequence confirmed these results [28]. High similarity between the analyzed mtDNA sequences of European mink and polecats (*M. putorius* and *M. eversmanii*) may be evidence of relatively recent speciation or gene flow through hybridization (reticulate evolution) occurring between these species [28,94].

**Figure 1:**
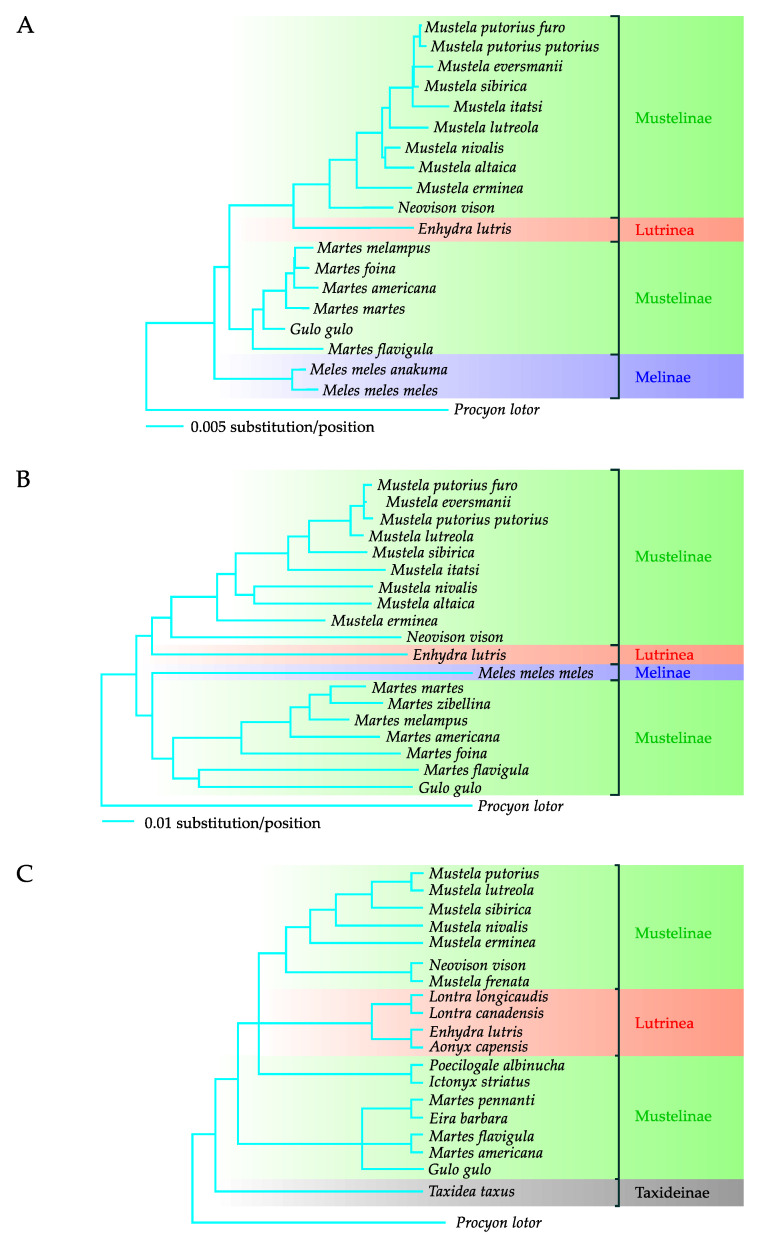
Dendrograms constructed for the Mustelidae family based on (**A**) nucleotide sequences (partial) of the *irbp* gene using the neighbor-joining method (the horizontal length of each branch is proportional to the number of nucleotide substitutions per site) [24], (**B**) complete nucleotide sequences of the *cytb* gene using the neighbor-joining method (the horizontal length of each branch is proportional to the number of nucleotide substitutions per site) [24], and (**C**) the sequences of *12S* rRNA, *cytb*, *ND2*, *tbg*, *irbp,* and *ttr* (first intron) genes using the maximum parsimony method with bootstrap support (1000 replicates) [31].

The phylogenetic analysis based on the nucleotide sequence of the nuclear *irbp* gene and the mitochondrial *cytb* gene, performed by Sato et al. [24], proved that *M. lutreola* belongs in the clade including European polecat, steppe polecat, Siberian weasel, and Japanese weasel (Figure 1A,B). The close evolutionary relationship between European mink, European polecat, and steppe polecat was also evidenced by the results of a phylogenetic analysis based on the sequences of mitochondrial genes *12S* rRNA [31,42,95] and *cytb* [22,25,31], and the gene encoding the NADH dehydrogenase subunit 2 [31], as well as the nuclear genes (the gene for thyroxine-binding globulin [31], *irbp* [31,95], the transthyretin-encoding gene [31], and the *Mel*08 complex repetitive flanking regions [25]). Sequences of *M. lutreola* nuclear genes (recombination activating gene 1, interphotoreceptor retinoid binding protein gene, apolipoprotein B gene, and transthyretin gene) were also used in a multispecies analysis of diversification timing of taxonomic groups within the Mustelidae family [96].

The multigene phylogenetic studies conducted by Flynn et al. [31] based on the fragments of sequence of three mitochondrial (*12S* RNA, *cytb*, and *ND2*) and three nuclear (*tbg*, *irbp*, and *ttr*) genes were particularly interesting. The results of these studies indicated that the European polecat is phylogenetically the closest species to European mink, whereas other closely related species include the Siberian weasel, the least weasel, and the stoat (Figure 1C).

According to phylogenetic analyses of Davison et al. [28] *M. putorius*, *M. eversmannii,* and *M. lutreola* should, despite the close evolutionary relatedness, be considered separate Evolutionary Significant Units (ESUs).

The phylogenetic distance between the European mink and the American mink is much greater than between *M. lutreola* and *M. putorius*, *M. eversmanii*, *M. nigripes*, *M. sibirica*, or *M. itatsi* [24,26,31,42,57,94,95,97,98]. American mink was separated from Eurasian representatives of the *Mustela* genus, including *M. lutreola*, and instead classified into the separate genus *Neovison* by Baryshnikov and Abramov in 1997 [99,100].

## 8. Molecular Ecology

Research concerning European mink in the field of molecular ecology, understood as the application of molecular genetic methods to address ecological questions [101], is mainly related to the ecology of pathogens (molecular epizootiology). Viral metagenomic analysis of European mink feces (collected in the Northern part of Spain), based on a random PCR in combination with next-generation sequencing, revealed the presence of genetic material of viruses from the following genera: *Amdovirus*, *Dependovirus*, *Parvovirus*, *Astrovirus*, and *Picobirnavirus* [34].

Recently, the nucleotide sequence of the angiotensin I converting enzyme 2 gene in *M. lutreola* was investigated to examine the potential of its protein product to be used as a receptor by the SARS-CoV−2 virus, and thus to verify if European mink could potentially be an intermediate host species for this pathogen [36]. The obtained results indicated very low predicted susceptibility of *M. lutreola* to SARS-CoV−2 [36].

Mañas et al. [102] detected the sequence of the *VP2* gene of the Aleutian mink disease virus (AMDV) using a PCR method in European mink from Spain. Leimann et al. [35] tested four individuals of *M. lutreola* from Hiiumaa Island (Estonia) for the presence of the AMDV DNA by PCR amplification of the *NS1* and *VP2* gene fragments. AMDV DNA was not detected in the examined animals [35].

An example of a bacterial pathogen identified in European mink using a PCR-Restriction Fragment Length Polymorphism (RFLP) method is *Borrelia burgdorferi*, which was the first report in this regard concerning animals originating in Romania [33]. Moinet [103] tested the renal samples of *M. lutreola* (Southwestern France) for the presence of pathogenic *Leptospira interrogans* DNA by PCR amplification. Of the 34 examined individuals, 8 were found to be infected with this pathogen, however, the role of European mink as a reservoir of *L*. *interrogans* was not confirmed [103].

In 2019, a pilot study on European mink detection and population monitoring using the environmental DNA (eDNA) metabarcoding method was performed in Northeastern Spain (La Roja and Basque Country) [62,63]. Due to its semiaquatic lifestyle, European mink DNA has a high detection potential from environmental (freshwater) samples, as is the case with other species living in aquatic environments [104,105].

## 9. Conservation Genetics

Although the number of studies on the genetics of European mink is relatively low, the important application aspect of almost every study in this field should be emphasized. Such studies directly contribute to obtaining valuable and practical knowledge about planning effective protective measures, both ex situ (conservation captive breeding) and in situ (reintroduction programs supplying the disappearing populations with individuals from the outside), conducted both ad hoc and as long-term strategies [7,66,89,106,107]. Conservation genetics can provide ready-to-use, measurable, and highly informative tools for defining conservation goals and means, which are crucial for the conservation of European mink [7,8,30,89,96,108]. Pioneering research, opening the chapter of European mink conservation genetics, was undertaken by Lodé [14].

The key issues for *M. lutreola* conservation to be addressed by conservation genetics are: (1) Determining the optimal species conservation scenario for management of the existing populations, including translocations and reintroductions (population restoration programs, repopulation); (2) captive breeding genetics; and (3) identification and assessment of possible threats to interspecies genetic nature, i.e., hybridization and introgression [7,8,66,108].

Since the publication of the first study results showing significant differences in genetic diversity between the Northeastern, Western, and Southeastern populations of European mink, the ESU concept [109] and definition of a management unit [110,111], which are useful for conservation purposes, represented hotly debated issues [89]. Originally, it was postulated to follow the precautionary principle, thus separately managing animals from the three geographically distinct populations [7,28]. The rationale for this approach was the risk of outbreeding depression [110], which is caused by individuals introduced from the outside and resulting in reduction in local adaptation [7]. Currently, the widely accepted interpretation of further genetic research involving larger samples and combined mitochondrial and nuclear markers is that European mink can be regarded as a single ESU and none of the three remaining populations demonstrate independent evolutionary development or a specific phylogeographic structure [1,8,30,112]. A critical assessment of this statement led to the assumption that, although the current large interpopulation genetic variation as a function of geographical distance does not automatically imply the phylogeographic structuring of the species (considering the time dimension apart from the geographical dimension), the currently observed genetic variation can be directly calculated (and may be only affected by measurement error), whereas the phylogeographic inference is indirect and secondary (and may be additionally affected by interpretation error).

The consequence of such reasoning is the acceptance of supplying the Western European ex situ stock with individuals from the Eastern European ex situ stock, exhibiting “better” values of the genetic diversity indicators [12]. Additionally, the translocation and (re)introduction of individuals from the captive Eastern population was suggested if inbreeding depression in French and Spanish wild populations were to be confirmed [2]. Cabria et al. [47] suggested the implementation of European mink conservation strategies that improve genetic connectivity by promoting gene flow among the scattered remaining populations. Restoration of reproductive exchanges was recommended by Lodé [14]. The European mink’s situation is basically similar to those of many other endangered mammal species in Western Europe, for which recovery projects mainly rely upon translocation of conspecifics from viable populations in Eastern Europe [113]. In planning, implementing, and evaluating the genetic management of *M. lutreola*, including reintroductions and translocations, experience in the conservation of closely related endangered species, e.g., the black-footed ferret, can be used [114,115,116,117].

The case of inbreeding depression is not simple and obvious—even if a loss of genetic variability is regarded as one of the major threats to European mink conservation [45], the low level of genetic diversity recorded in the French and Spanish populations being natural and not impairing species plasticity in habitat use and colonizing capacity cannot be ruled out (the so-called “mink paradox”) [14,118]. The genetic consequences of range expansion, such as the structuring of newly colonized areas into distinct sectors of low genetic diversity [119] as well as the phenomenon of heterozygosity excess following bottleneck events lasting for several generations [120], seemed to be overlooked by authors who interpreted the low genetic variation in populations from France and Spain. However, Carbonell [89], after Dlugosch and Parker [121], concluded that losses of quantitative variation in expanding populations may be minimal compared with losses of molecular variation.

Considering the historical range extent of European mink, an interesting colonization scenario hypothesis, according to which the French population of *M. lutreola* originates from the Black Sea area, was formulated by Lodé [118]. According to this hypothesis, the expansion occurred along the Danube, passing through the north of the Alpine arc and following the Loire River toward Western France [118].

The potential risk of outbreeding depression (which is connected with translocations between populations) as noted previously, needs to be further investigated and assessed [8,28,30,122]. Increasing the number of samples and broadening their geographical representation, as well as increasing the number of analyzed sequences (including genomic analysis), are not substitutes for a comparative analysis of intraspecies genetic diversity in the historical dimension. Such research requires a comparative analysis of samples from contemporary and historical populations (aDNA obtained from fossils and museum specimens, natural history collections), including those from areas where the species is no longer present. Inferences about the causes and effects of the currently observed genetic variation in European mink without historical comparative analysis would be speculation. The problem regarding the lack of comparative aDNA material was reported by Cabria et al. [30]. Knowledge of the original resources of genetic diversity would allow proper assessment of the present state of the species’ gene pool, providing baseline levels of diversity, inbreeding, and genetic load [117,123]. This knowledge can further be used to predict how organisms might respond to human-induced global change in the future [124].

Historical material, although on a limited scale covering the period 1983–2006, was included in the analysis of genetic variation conducted by Korablev et al. [83] for the European mink population in the Central Forest Reserve (Tver oblast) in Russia. The authors concluded, based on the results of genetic and morphometric (dynamics of odontological and craniological characteristics) analyses, that the catastrophically rapid extinction of *M. lutreola* in the study area, preceded by a period of population insularization, led to a gradual reduction in its phenetic polymorphism, albeit without major loss of genetic diversity [83].

For management units, since the results of the conducted research did not allow for their definition, the concept of a distinct population segment (DPS) was proposed to apply to European mink [7,8,125]. According to Michaux et at. [8] and Cabria et al. [30], the remaining three main populations of European mink cannot be considered DPSs, as genetic variation was not found to be geographically structured among the three European populations of *M. lutreola*. Nevertheless, the results indicated highly significant differences in parameters describing the genetic diversity between all three populations of European mink [7,8,28,29,30,45,49,85]. A review of research showed that promotion of the pragmatic interpopulation translocations should not be justified by misinterpretation of the results of genetic analyses, but rather by the need to choose the lesser evil in the critical situation of a species experiencing extinction before our eyes. A lesser evil in this context means assuming the risk of outbreeding, losing the geographical originality of the population gene pool, and the potential loss of local adaptations.

Important aspects for in situ conservation of wild populations are the demographic indicators that describe the conditions needed to retain genetic diversity in the population and determine the potential survival of endangered species, i.e., effective population size (*N_e_*), which determines heterozygosity decreases due to genetic drift at a rate of 1/2*N_e_* per generation, and genetic neighborhood area (*N_a_*), which represents the area within which adults can randomly mate [15,126,127,128]. Both indicators were estimated for the population of European mink from Southwestern France (Charente-Maritime) by Lodé and Peltier [15] to assess how its decline affected the ability to retain genetic diversity in the population. The calculated value of *N_a_* was a diameter of 31.7 km, allowing for population size within the neighborhood area to range from 16.1 to 22.8 individuals [15]. The *N_e_*/*N* ratio, where *N* is the adult population size, ranged from 0.089 to 0.197 for the studied population [15], which is close to the average for wildlife populations, as reported by Frankham [129]. The estimated value of the genetic neighborhood area provides a guide for the designation of protected areas dedicated to European mink conservation [15].

Although the abovementioned achievements of population genetics, phylogenetics, phylogeography, studies on genetic markers, identification by genetic methods, and molecular ecology of European mink focused on wild populations and their conservation in situ, captive breeding genetics addresses the issues of maintaining and developing captive stocks to preserve the species’ genetic pool. Captive (or conservation) breeding genetics can be defined as a subfield of conservation genetics that aims to provide genetic theory and techniques for conservation breeding of captive wildlife stocks before the reintroduction (repopulation) phase can be achieved. The key issues covered by captive breeding genetics for *M. lutreola* include the selection of individuals intended for mating (reproduction), preventing inbreeding and genetic erosion in ex situ stocks, and studying the genetic basis of traits that are important for reproduction in captivity [130].

The first conservation breeding program for European mink was launched in the Tallinn zoo (Estonia) in 1984; the European Endangered Species Program (EEP) for the species was launched by the European Association of Zoos and Aquaria (EAZA) in 1992 [4,66]. The captive stock comes from 22 founders, mostly originating from Northeastern and central Russia, and currently includes 220 individuals [12]. The total European mink ex situ EEP population is 267 [12]. Animals are kept in 26 breeding facilities in Czechia, Estonia, Finland, France, Germany, Latvia, the Netherlands, Poland, and Slovakia [66]. A regional captive breeding program was also initiated in Spain in 2004, with 10 founders captured in the country [66]. Currently, about 47 breeding individuals are held in nine breeding centers in Spain [12]. Since 2010, breeding of European mink was carried out in the European Mink Breeding Centre of the Ilmen Nature Reserve (Chelyabinsk oblast, Russia) [131]. The 30 founders of this captive stock were from the Institute of Systematics and Ecology of Animals of the Siberian Branch of the Russian Academy of Sciences in Novosibirsk (Russia), where European mink was first bred in captivity in the 1970s and continues to be bred here [132]. There are also plans to establish an ex situ breeding center in Romania [12].

The conservation goal of the captive breeding programs for *M. lutreola* is to preserve the genetic diversity of the species in captivity, as well as supply individuals for reintroduction [133]. They also play an important role in research applications in the field of conservation genetics [12,134]. Captive-born animals from the EEP program are used for reintroduction purposes in Estonia and Germany, whereas reintroductions in Spain use individuals born under the Spanish captive breeding program [66,135]. Specifically, the EEP program for *M. lutreola* aims to maintain 97.5% of the original genetic diversity of the founders in an ex situ population for as long as possible [12].

Effective population size for the Eastern ex situ population was calculated as 65.6 individuals [133]. Maran et al. [12] reported the values of the following genetic indicators for the captive population, namely, founder genome equivalents (*FGE*; the number of wild-caught individuals/founders that would produce the same amount of gene diversity as does the population under study), (retained) genetic diversity (*GD*; the probability that two alleles from the same locus sampled at random from the population will not be identical by descent), population mean kinship (*MK*; the proportional loss of gene diversity of the descendant captive-born population relative to the founders and to the mean inbreeding coefficient of progeny produced by random mating), mean inbreeding (*I*; the probability that the two alleles at a genetic locus are identical by descent from an ancestor common to both parents), and effective population size/census size ratio (*N_e_*/*N*). The values of the indicators for the Eastern (EEP) and Western (Spanish) European captive populations were: *FGE,* 7.3 and 1.18; *GD,* 93.2% and 57.5%; *MK,* 0.068 and 0.425; *I,* 0.079 and 0.364; and the *N_e_*/*N* ratios were 0.283 and 0.073, respectively [12].

Conservation breeding programs are characterized by the selection of the genetically most suitable pairs for reproduction, identified according to their genetic representation among the population to avoid inbreeding [136]. A common problem is that the specifically selected partners for breeding do not mate during the breeding attempts [136]. Kneidinger et al. [136] reported that, on average, just over 30% of the planned litters were sired by the genetically prioritized males. The influence of selection on the behavioral traits of breeding males on shaping the gene pool in captivity is not yet recognized. Aggressive behavior by the male toward the female excludes the former from mating, but without knowledge of the possible heritability of personality traits, it is not possible to assess the risk of decreasing genetic variation in the reintroduced populations posed by selection for release of individuals with specific personality types [136,137,138]. Conservation genetics can help to gain further insight into the impact of genetic factors, which, in addition to environmental conditions, cause the low breeding success of *M. lutreola* individuals in captivity [133].

Conservation breeding of *M. lutreola* in captivity may be enhanced by implementation of assisted reproductive techniques (ARTs), including semen and embryo cryopreservation, artificial insemination, and embryo transfer [12,134,139,140,141]. The use of ART, however, requires analysis of their potential impact on the genetic parameters of the offspring (e.g., as reviewed by Lin and Tsai [142]), and there are no reports of such studies for European mink in the available literature. Development of protocols for European mink sperm cryopreservation and biobanking of DNA samples and other biomaterials (genome resource banking (GRB)) to facilitate future research and interventions is planned under the EEP program dedicated to this species [12]. Biobanked semen, or even fibroblast cell lines, could be used to revive genetic diversity that was lost over time [143].

The role of captive breeding of European mink is of critical importance for the survival of the species. However, per Ehrlich [144], “the loss of genetically distinct populations within species is, at the moment, at least as important a problem as the loss of entire species”. Therefore, efforts to conserve the genetic resources of the preserved wild populations of *M. lutreola* in situ cannot be overestimated. Conservation genetics can provide knowledge that could be applied to reorient conservation strategies to prioritize the survival of threatened distinctive populations [113]. Appropriate guidance can be provided by the lesson learned from the *M. nigripes*, *M. eversmanni*, and *M. putorius* conservation breeding and reintroduction program [116,117,143,145,146,147,148,149]. When a species is threatened with extinction, the timing of measures to conserve genetic resources is of particular importance. Due to the decreasing sizes of the preserved populations (resource constraints increasing over time) and the increasing risk of inbreeding depression limiting natural breeding success, GRB and ART programs need to be initiated before genetic health issues arise [143].

It was claimed that genetically managed, long-term breeding programs within zoological gardens can be a source of individuals for reintroduction [150]. However, the question still remains regarding the differences in genetic diversity of wild and captive populations, regardless of their geographical origin, as European mink may be subject to reduced natural selection pressure in captivity [50]. Becker et al. [50] stated that this aspect should be considered whenever founder individuals for breeding and reintroduction are selected. Selective conservation breeding may lead to an increase in inbreeding, resulting in genome-wide loss of variation [151].

Two important issues addressed by conservation genetics include whether the risks of hybridization and introgression exist for European mink and, if so, the size of these risks. The close phylogenetic relationships between European mink and European polecat were proven by the lack of a complete interspecies reproductive barrier between these taxa, expressed by spontaneous hybridization in nature [28,47,49,152]. Davison et al. [28] indicated that at least one mtDNA haplotype was shared between both species, suggesting mitochondrial introgression. The hybridization is asymmetric, as *M. putorius* males mate with *M. lutreola* females [2]. The number of chromosomes (*2n*) in hybrids can be 39 or 40 [1]. The frequencies of hybridization and genetic introgression in natural conditions are low, estimated at ≤3% [47,49] and 0.9% [47], respectively. Even if natural hybridization events occur only occasionally, interspecies gene flow can play some role, especially in accelerating local extinction of declining and fragmented populations, e.g., by outbreeding depression [1,28,47,49,153].

Identification of hybrids using genetic methods is important for captive breeding, as using hybrids in the ex situ breeding programs is not advisable [153]. Potential for hybridization and its management should also be considered in reintroduction planning and implementation, as it poses a risk to the genetic integrity of the reintroduced populations [154,155]. Lodé et al. [49] described a method of discrimination between European mink, European polecat, and their hybrid based on differences in five allozymic and four microsatellite diagnostic loci (Table 4). The hybrid showed an intermediate pattern for microsatellite markers [49].

Analyses conducted by Cabria et al. [47] involved 317 European mink, 114 polecats, and 15 putative hybrids from different localities in Europe. Assigning individuals to species (or hybrid group) was based on genotyping with 13 microsatellite nuclear markers (Table 4) and Bayesian analysis of biparental multilocus genotypes employing clustering based on allele and genotype frequencies. Private haplotypes exhibiting high discriminating power accounted for 34.33% and 27.03% of all identified haplotypes in European mink and European polecat, respectively, while most alleles shared by these species showed differences in the distribution of allele frequency between species [47]. Of 14 detected hybrids, 12 were from French and Spanish populations [47].

Hybridization direction (paternal and maternal origins) was detected by direct nucleotide comparison of a sequenced 614 bp mitochondrial control region and partial sequences of introns 5 and 7 of the *DDX3Y* gene located on the Y chromosome [47]. The obtained results proved that hybridization is asymmetric, as only pure European mink females mated with pure European polecat males, and backcrossing and genetic introgression only occurred from female first-generation (*F*1) hybrids of *M. lutreola* to *M. putorius* [47]. An important implication of the described research was that the continuing decline of European mink may be associated with the growing significance of hybridization as one of the most important threats to the survival of the species due to the increasing avoidance of conspecific mates [47]. Additionally, the identified asymmetry in the hybridization process may, given the progressive decline of remaining populations, lead to a progressive assimilation (introgression) of European mink by European polecat [47,49].

The possibility of crossbreeding between European mink and the ferret *M. putorius furo* [156,157] was also reported. No possibility of crossbreeding between European mink and American mink [157,158] or Siberian weasel [159] was proven. Hybrid embryos for of these species obtained in laboratory conditions exhibited pathological characteristics and were resorbed in a short time [158,159].

The final reflection on the European mink conservation genetics may be a reference to the definition of this subdiscipline of genetics, namely, a reminder that the imperative, apart from preserving the natural intraspecies genetic diversity, is to ensure the continuity of evolutionary and ecological processes responsible for the formation and maintenance of this diversity [32].

## 10. Conclusions

The overall conclusion from the analysis of the history and scope of genetic research on European mink is a gradual increase in the genomic representativeness of the nucleotide sequences used. This trend is in line with the widespread transition from genetics to genomics, which is also (and perhaps especially) observed in conservation genetics [160,161,162]. Important in the case of *M. lutreola*, advanced genomics (whole-genome and reduced-representation approaches) can help to describe and explain adaptive genetic variations, resolving phylogenetic and phylogeographic questions, identifying adaptive alleles, as well as identifying and quantifying inbreeding, hybridization and introgression in a more accurate way than genetics [163,164,165,166,167]. Whole-genome sequencing also provides valuable knowledge about the origin and evolutionary history of endangered species and defines units for conservation, thus helping to improve conservation strategies [165,166]. For a nonmodel endangered species, such as European mink, obtaining a reference genome is essential [168,169].

Genomic methods considerably improved conservation efforts toward some mammalian endangered species. The whole-genome approach was previously applied to the conservation of African wild dog *Lycaon pictus* to detect inbreeding and population-specific selection [170]. Genomics provides tools for population evaluation monitoring and management of small populations in the wild and in captivity, species delineation, and to enhance wildlife health management and identify risk factors for genetic disorders of endangered primates [171,172,173]. Conservation genomics helped to identify the conservation implications of admixture in the Eastern wolf *Canis lupus lycaon* [165]. Wright et al. [174] identified several candidate genes that may be associated with variation in the breeding success of the Tasmanian devil *Sarcophilus harrisii*. From the *M. lutreola* population genetics perspective, the same research demonstrated that individual heterozygosity was not associated with breeding success in captivity but was negatively associated with litter sizes of breeding females [174].

The correct selection of an appropriate sample size for population genetics research seems to be a neglected issue. Determination of the optimal level of sampling effort required for adequate characterization of the intraspecies genetic variation is of fundamental importance [175]. Too many samples than required for accurate estimation of genetic diversity increases costs and workload and lengthens the analysis, whereas too small a sample size results in significant errors in estimating the genetic diversity [176,177,178]. Particular sensitivity of mtDNA and microsatellite markers to sample sizes in addressing questions related to interpopulation genetic diversity, phylogenetics, and phylogeography leads to recommendations of using genomic data over microsatellites or a limited number of mitochondrial or/and nuclear single nucleotide polymorphism markers for genetic studies [175,179,180,181]. The limited availability of samples for genomic studies on *M. lutreola* also needs to be further investigated, as sampling size may be a significant limitation of genomics in conservation [166].

In future research on the genetic diversity of the surviving European mink populations, much more attention should be paid to optimization of the sample size, as sampling many individuals per population but using a small number of genetic markers does not contribute to accurate and reliable results [178,182]. Genomics offers a solution: Genome-wide techniques, such as restriction site-associated DNA sequencing (RAD-seq), to acquire a large number of single nucleotide polymorphisms (SNPs), allowing finer identification of population structure and stronger determination of patterns of isolation-by-distance than with microsatellites, with a smaller sample size [164,183]. The need to address the small number of available samples is crucial given the declining wild populations of European mink. Obtaining genetic samples from the rediscovered, relic wild populations of the species in the Dniester Delta in Ukraine is also important as a completely recently rediscovered Caucasian population or a presumptive Carpathian population [2,184,185]. The application of eDNA-based species detection is promising in rediscovery research projects.

Based on the analysis of the research work completed to-date in the field of *M. lutreola* genetics, the following research issues remaining to be addressed and resolved are as follows:

Research Issues Per Se:

1. Establishing a karyotype reference standard;
2. Initiation and completion of the whole-genome sequencing project (an improved scaffolded genome of *M. putorius* GenBank assembly accession GCA_902207235.1, and a platinum quality genome available for *M. erminea*, RefSeq assembly accession GCF_009829155.1, could be used for reference-based assembly or designing primers for any genomic location for targeted sequencing);
3. Development of a genome-scale (mitogenome-scale) SNPs panel, optimal for the study of inter- and intrapopulation genetic diversity, and the species phylogeny (possible revision of taxonomic status at the genus level) and phylogeography.

Conservation Issues:

4. Resolving the issue of undertaking conservation actions (including translocations) of wild persisting populations (scientifically-informed decisions regarding whether to treat them as a single or separate management units, which is especially relevant given the plans for the inclusion of the Spanish breeding program in the EEP program and plans for obtaining new founders from the wild Romanian population [12,164]);
5. Assessment of the impact of the breeding process on the development of traits essential for survival in the wild (adaptation to captivity) and the role of (re)introduced individuals in shaping the gene pools of wild populations (potential outbreeding and loss of unique adaptations, which could be assessed using captive-breeding experiments, in which individuals from distinct populations are hybridized to check if a loss of fitness is occurring);
6. Phylogeographic reconstruction in terms of origin of the French–Spanish population.

Promotional and Organizational Issues:

7. Encouraging the scientific community to undertake research related to European mink genetics, as well as its promotion and popularization (e.g., in the context of scientific social responsibility (SSR));
8. Scientific cooperation and networking (including sharing experiences, sharing samples, mentorship, and transfer of research results to conservation practice);
9. Development of protocols for the preservation and biobanking of the species’ genetic resources, which could be based on the extensive achievements and experience in this field with the Black-footed ferret [12,117,143].

Maran et al. [12] listed the molecular genetic studies linked to the long-term management plan for European mink aiming to (1) determine if the population introduced onto Hiiumaa Island (Estonia) is in need of genetic supplementation in the coming years, (2) study the genetic diversity of the founders originating from the Western European population and the wild Eastern European population to determine whether this follows the same pattern found in earlier studies, and (3) encourage interest in the genome sequencing of European mink.

The question of the potential influence of reduced genetic diversity (e.g., in terms of MHC [50]) on the viability and survival of the wild and reintroduced populations also remains unresolved. Research initiatives should be undertaken to help resolve how genome alteration in each demographic influences *M. lutreola* species viability in a global change scenario [125,164].

Just as important as experimental research are reviews and (meta-)analytical works that indicate how gained genetic knowledge can be used to solve specific problems facing the conservation of *M. lutreola*, thereby indicating the application value of this knowledge.

The initiative to establish the European Mink Centre (www.europeanminkcentre.org) is worth mentioning; an internet platform, acting as a digital repository, information hub, and think tank dedicated to conservation biology, including conservation genetics, of the species [186]. The Centre’s aim is to stimulate and facilitate research in the field of *M. lutreola* conservation genetics [186]. The promotion of the achievements of conservation genetics is of particular importance in the European mink case, as the success of the implemented conservation measures depends to a large extent on the favor of decision-makers and the general public. Hence, initiatives such as the European Mink Day proposed in 2015 by the Polish Society for Conservation Genetics LUTREOLA on 31 March may have an indirect and positive impact on creating research interest in *M. lutreola* and implementation of genetic research results in its conservation [187].

## Figures and Tables

**Table 1:** Microsatellite loci identified for *Mustela lutreola* by Cabria et al. [9].

Microsatellite Loci	GenBank Accession Code	Repetitive Motif	Number of Alleles Identified
*Mlut*04	EF093582	(GT)_16_	5
*Mlut*08	EF093583	(GT)_12_	4
*Mlut*15	EF093585	(GT)_14_	5
*Mlut*20	EF093587	(GT)_18_	8
*Mlut*25	EF093588	(GT)_15_	6
*Mlut*27	EF093589	(GT)_8_NN(GT)_14_	2
*Mlut*32	EF093590	(GT)_59_	8
*Mlut*35	EF093591	(GT)_15_NNNN(GT)_4_NN(GT)_7_	4

**Table 2:** Microsatellite loci identified for *M. lutreola* by Cabria et al. [9].

Species	Microsatellite Loci	Reference
American mink	*Mvi*002, *Mvi020*, *Mvi*022, *Mvi*054, *Mvi*072, *Mvi*075, *Mvi*111, *Mvi*389, *Mvi*1843	Michaux et al. [8], Cabria et al. [30], Peltier and Lodé [45], Lodé et al. [49]
European polecat	*Put*FK1	Peltier and Lodé [45]
Stoat	*Mer*009, *Mer*022, *Mer*041	Michaux et al. [8], Cabria et al. [30]

**Table 3:** Level of similarity (max identity parameter) between the complete sequence of the mitogenome of European mink and selected species of the family Mustelidae (developed using the BLAST program [59]).

Taxon	Similarity [%]	Taxon	Similarity [%]
*Mustela putorius*	99	*Enhydra lutris*	87
*Mustela putorius furo*	99	*Lutra lutra*	87
*Mustela evermannii*	99	*Lutra sumatrana*	86
*Mustela nigripes*	98	*Martes melampus*	86
*Mustela sibirica*	97	*Martes Americana*	86
*Mustela itatsi*	95	*Martes martes*	86
*Mustela altaica*	92	*Martes zibellina*	86
*Mustela nivalis*	92	*Martes flavigula*	86
*Mustela ermine*	92	*Martes foina*	86
*Mustela kathiah*	89	*Martes pennant*	86
*Mustela frenata*	89	*Gulo gulo*	86
*Neovison vison*	88	*Melogale moschata*	86

**Table 5:** Indicators of genetic variety of the Northeastern (NE), Southeastern (SE), and Western (W) populations of European mink, based on 11 microsatellite loci [30].

Population	*N*	*N_A_*	*P_A_*	% *P_A_*	*A*	*H_O_*	*H_E_*	*F_IS_*
NE	107	59	20	33.90	5.364	0.559 ± 0.153	0.613 ± 0.164	0.089
SE	44	35	2	5.71	3.182	0.464 ± 0.170	0.496 ± 0.139	0.065
W	162	32	3	9.38	2.909	0.336 ± 0.161	0.439 ± 0.201	0.236
TOTAL	313	64	-	-	5.818	0.430 ± 0.113	0.578 ± 0.148	0.255

*N*, number of examined individuals; *N_A_*, number of alleles identified; *P_A_,* number of private alleles; % *P_A_*, percentage of private alleles in total number of alleles; *A*, allelic diversity; *H_O_*, observed heterozygosity; *H_E_*, expected heterozygosity; *F_IS_*, inbreeding coefficient.
